# Identification of Volatile Organic Compounds and Analysis of Aroma Characteristics in Ten Pear Syrups

**DOI:** 10.3390/foods13203223

**Published:** 2024-10-10

**Authors:** Yang Wang, Wei Tong, Wenhui Wang, Yanmin Du, Xiaohui Jia, Zhihua Wang, Jianyi Zhang, Hailong Sun

**Affiliations:** 1 Institute of Pomology, Chinese Academy of Agricultural Sciences, Xingcheng 125100, China; wangyang@caas.cn (Y.W.); wangwenhui@caas.cn (W.W.); duyanmin@caas.cn (Y.D.); jiaxiaohui@caas.cn (X.J.); wangzhihua@caas.cn (Z.W.); zhangjianyi@caas.cn (J.Z.); sunhailong@caas.cn (H.S.); 2 Key Laboratory of Germplasm Resources Utilization of Horticultural Crops, Ministry of Agriculture and Rural Afffairs, Xingcheng 125100, China; 3 Key Laboratory of Fruits Storage and Processing of Liaoning Province, Xingcheng 125100, China

**Keywords:** volatile organic compounds, pear syrup, OPLS-DA, OAV, HS-SPME, GC-MS

## Abstract

Aroma in food plays an important role in food perception and acceptance, which depends on various mixtures of volatile organic compounds (VOCs). Moreover, VOCs are of great significance for aroma identification. In this study, headspace solid-phase microextraction (HS-SPME) combined with gas chromatography–mass spectrometry (GC-MS) technology was used to determine the VOCs in 10 pear syrups. A total of 127 VOCs were quantitatively determined, including 9 common VOCs and 46 characteristic VOCs of 10 pear syrups. The pear syrups were divided into three categories by cluster analysis, and thirty-eight differential VOCs were obtained using orthogonal partial least squares discrimination analysis (OPLS-DA) and fourteen key VOCs were selected by odor activity value (OAV). It was revealed that the key and common aroma components of pear syrups were butanoic acid, methyl ester, 2-methyl-, methyl ester and Hexanoic acid, and ethyl ester. The characteristic and differential VOCs were 10-Undecen-1-ol, Hexadecanal, n-Propylacetate, Cyclohexanol, 5-methyl-2-(1-methylethyl)-, (1S,2R,5S)-, Methional, Disulfide, dimethyl, 8-Nonenoic acid, ethyl ester, Naphthalene, 1,2-dihydro-1,1,6-trimethyl-, 3H-Purin-6-amine, N,N,3-trimethyl-, 2-Octanol,2,6-dimethyl-, Furyl hydroxymethyl ketone, Heptane, 2,2,4,6,6-pentamethyl-, and Butanoic acid,2-methyl-,methyl ester. The above results showed that different pear syrups had rich diversity in aroma compounds, with some components being shared among them while others are exclusive to specific syrups.

## 1. Introduction

China holds the highest area and production for pear cultivation in the world. In 2022, the global annual output of pear fruit was 26.32 million tons [1]. China is home to an extensive assortment of pear types, boasting a collection of more than 2,000 preserved varieties [2]. As a fruit and traditional medicine, pear fruit has anti-inflammatory, cough, and diuretic effects [3,4]. Recently, pear-based processed products have received considerable attention owing to their mitigative action against the surge in pneumonia, asthma, cough, and some diet-related ailments [5,6]. Pear syrup is a popular local dish in Asia, especially in China. The process of making pear syrup by carefully pressing and sifting first. Afterward, the extracted juice is gradually heated. This slow heating continues until the soluble solid content increases to approximately 80 °Brix. As the process advances, the color of the syrup deepens, eventually turning a rich, dark brown [7].

Aroma profoundly affects both the level of consumer satisfaction and the sensory quality of food. Aroma represents a complex blend of diverse volatile organic compounds (VOCs), including esters, alcohols, aldehydes, ketones, terpenes, hydrocarbons, acids, etc. [8,9,10,11,12]. Preserving the original flavor during food processing is challenging and costly, resulting in expensive natural flavor products [13]. People often add flavoring by adding essence. Therefore, appropriate analytical methods are particularly important for food aroma quantification [14]. In the realm of metabolomics, the analysis of volatile organic compounds (VOCs) plays a crucial role. It is highly significant for monitoring and identifying food substances. This analytical approach aids in tracking and determining the specific characteristics of various food items [15]. The initial step involves collecting VOC information and screening out the signature substances. Gas chromatography-mass spectrometry (GC-MS) coupled with headspace solid-phase microextraction (HS-SPME) is an uncomplicated, rapid, and inexpensive technique that does not require organic reagents [16,17,18]. This method has found extensive application in detecting VOCs across various fruits, including apples [19], pears [20], blueberries [21], and other fruits [22,23].

In this study, pear syrups made from different pear fruits were used as raw materials to analyze the difference in their VOCs. A large number of scholars found that there were differences in VOCs in different pear fruits [8,20,24,25,26], but there was no report on the analysis of VOCs in pear syrup. In this paper, the composition of VOCs in 10 pear syrups was analyzed. The common and distinctive VOCs that characterized pear syrup were screened out. The differential and key VOCs of each pear syrup can be used as identification markers. In addition, the findings can provide a data basis for the identification and production of pear syrup.

## 2. Materials and Methods

### 2.1. Reagents and Chemicals

The internal standard, 3-Nonanone (>99%), was sourced from Sigma-Aldrich (Titan Co., Ltd., Shanghai, China). A 10% methanol solution was used to prepare the standard. The methanol, which was of HPLC grade, was procured from Fisher Scientific (Pittsburgh, PA, USA). Purified water was supplied by Wahaha Foods Co., Ltd. (Hangzhou, China). Sodium chloride (NaCl) was provided by Macklin Co., Ltd. (Shanghai, China).

### 2.2. Sample Preparation

Ten pear syrups were obtained through Taobao, Jingdong and offline supermarkets, respectively. Samples with only pear ingredients on the ingredient list were selected in Table 1. Moreover, images of the pear syrups can be found in Figure S1. The preparation of the sample was carried out under ambient conditions, maintaining a temperature of approximately 20 °C throughout the process. Briefly, 4 g of pear syrup was mixed with 20 mL of distilled water (Figure S2). Subsequently, 2.5 mL of the above solution was incorporated with 0.9 g Nacl and 50 μL 0.04 g/L standard solution of 3-Nonanon. The mixture was transferred into an SPME vial. Each sample was collected in triplicate.

### 2.3. HS-SPME Conditions

To extract VOCs, a specialized SPME fiber made of divinylbenzene, carboxin, and polydimethylsiloxane (DVB/CAR/PDMS) 50/30 µm thick was employed. This fiber, acquired from Sigma-Aldrich (Supelco, Bellefonte, PA, USA), was utilized in the extraction process. The procedure involved the AOC 6000 auto-sampler, a precision instrument manufactured by Shimadzu (Tokyo, Japan), to ensure accurate sample handling. Prior to each analytical session, the fiber underwent a conditioning step, where it was heated to 250 °C for a period of 10 min. To improve the VOCs’ dissociation, the samples were then incubated for 15 min at a controlled temperature of 45 °C. After this incubation, the conditioned fiber was placed into the vial’s headspace, where it was left for an additional 15 min. During this period, the VOCs were extracted while the agitator was set to operate at 300 rpm, maintaining the system at the incubation temperature of 45 °C.

### 2.4. GC-MS Analysis

The samples were subjected to analysis utilizing a 2010plus GC, which was paired with a QP2020 mass spectrometry system, both produced by Shimadzu (Tokyo, Japan). To desorb the VOCs absorbed on the SPME fiber, the injector port was heated to 200 °C, and the desorption process was carried out for 1 min under splitless mode. The gas chromatograph was equipped with an HP-INNOWAX capillary column from Agilent Technologies (60 m × 0.25 mm × 0.25 µm, Santa Clara, CA, USA). The initial column temperature began with an initial setting of 40 °C, which was maintained for 1 min. Subsequently, the temperature was raised by 2 °C per minute until it reached 200 °C, where it was held for 1 min. Following this, the temperature was further elevated at a rate of 10 °C per minute until it reached 230 °C, with this final temperature being held for 10 min. Helium, with a purity of 99.999%, was employed as the carrier gas and was kept at a constant flow rate of 1.0 mL/min.

MS analysis was conducted under the electron ionization (EI) mode, utilizing an ionizing energy of 70 eV. The detector’s ion source was maintained at a temperature of 230 °C. During the analysis, mass spectra were scanned over a range of 50–500 *m*/*z*. To identify these compounds, spectral data were compared against the NIST 11s library database, with a requirement that the spectral similarity exceeded a matching quality of 90%. Additionally, match quality per VOC peak was meticulously verified.

### 2.5. Data Processing and Statistical Analysis

#### 2.5.1. VOC Content

Quantification of VOCs was achieved through the use of the internal standard method. In this approach, the concentration of each compound was adjusted relative to 3-Nonanone. The peak areas, essential for the quantitative analysis, were determined by integration. VOC quantification was carried out using the following equation:

$$C_{v}=\frac{\frac{A_{v}}{A_{i}}\times C_{i}\times V_{i}\times M_{i}}{M\times M_{j}\times 10^{-3}}$$

*C*_v_ is the content of VOCs (μg/kg FW); *A*_v_ is the peak area of VOCs; *A*_i_ is the peak area of the internal standard; *C*_i_ is the concentration of the internal standard (0.04 g/L); *V*_i_ is the volume of the internal standard (50 μL); *M*_i_ is the mass of the diluent (24.0 g); *M*_j_ is the mass of the diluent sample (2.5 g); and *M* is the mass of the sample (4.0 g).

#### 2.5.2. Odor Activity Values (OAVs)


$$\mathrm{OAV}=\frac{C_{v}}{O_{T}}$$

*C*_v_ is the content of VOCs (μg/kg FW) and *O*_T_ is the oder threshold (μg/kg).

#### 2.5.3. Multivariate Statistics

For data analysis and graphic presentation, Excel 2010 (Microsoft, Redmond, WA, USA) software was employed. An upset map was created using online analysis software (https://www.chiplot.online) (accessed on 18 March 2024). To explore clustering patterns and establish connections between cultivars and VOCs, principal component analysis (PCA) and cluster analysis were carried out with Origin 2021 (MathWorks, Natick, MA, USA). Additionally, orthogonal partial least squares discriminant analysis (OPLS-DA) was executed utilizing SIMCA version 14.0 (Umetrics, Umea, Sweden) and R (version 3.5.1).

## 3. Results

### 3.1. Identification and Quantification of VOCs

**Figure 1:**
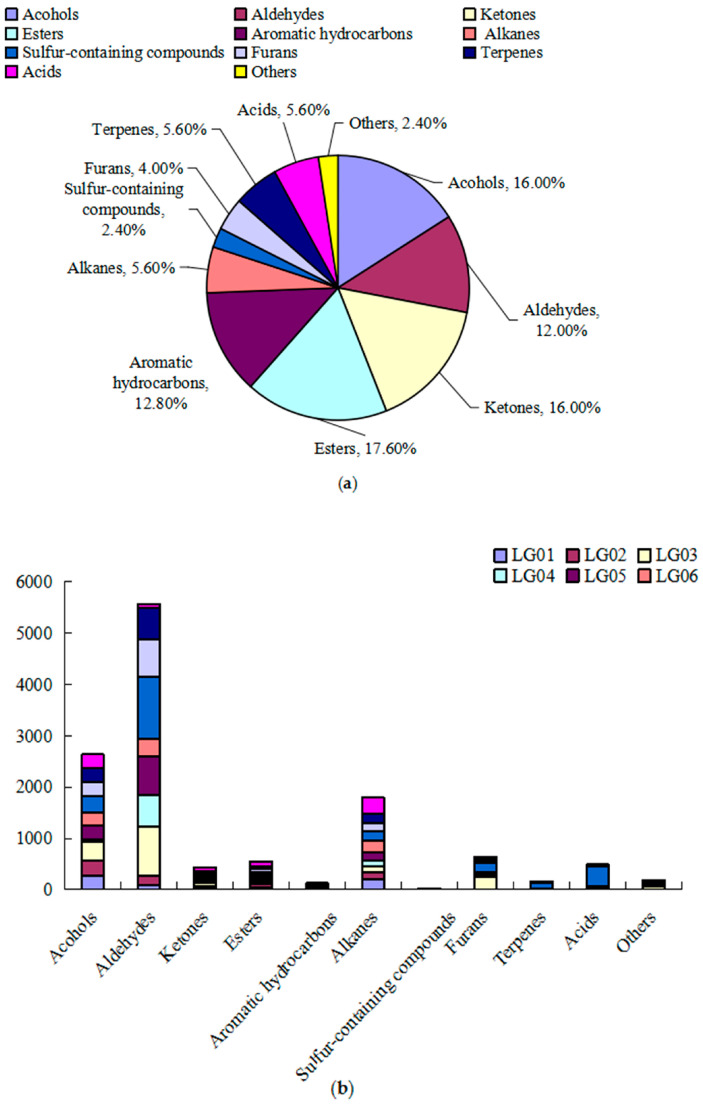
General classification diagram (**a**) and comparison diagram of various component content (**b**) in 10 pear syrups.

A total of 127 VOCs and 11 categories were detected through the analysis of volatile components in 10 pear syrups, with each peak identified and classified using spectral library search (Table 2 and Figure S3). Ester compounds were the most abundant, comprising 22 species, which accounted for 17.19% of the total species quantity, followed by alcohols and ketones, each with 20 species, making up 15.63% of the total species quantity. Aldehydes and aromatic hydrocarbons represented 18 and 16 species, respectively, accounting for 14.06% and 12.50% of the total species quantity. Other compounds, such as alkanes (seven species), sulfur-containing compounds (three species), furans (four species), terpenes (seven species), acids (seven species), and others (three species), had less than ten species each (Figure 1a).

The analysis of different categories of VOCs (Figure 1b) revealed that aldehydes had the highest total content at 5572.89 μg/kg FW, representing 43.65% of the total content, followed by alcohols, with a content of 2892.73 μg/kg FW, representing 22.66% of the total content, and alkanes, with a content of 1793.42 μg/kg FW, making up 14.05% of the total content. The proportions of the remaining categories were less than 10%. Aromatic hydrocarbons and sulfur-containing compounds had the lowest contents, with 126.60 μg/kg FW and 8.95 μg/kg FW, respectively. It was evident that aldehydes, alcohols, and alkanes were the primary VOCs of 10 pear syrups.

The VOCs of 10 pear syrups were analyzed, revealing that LG07 had the highest cumulative content of VOCs at 2575.64 μg/kg FW, followed by LG03 (1894.61 μg/kg FW), LG04, LG05, LG08, and LG09, which exhibited intermediate levels with contents of 1153.74 μg/kg FW, 1341.93 μg/kg FW, 1313.60 μg/kg FW, and 1274.24 μg/kg FW, respectively. On the other hand, LG01, LG02, LG06, and LG10 displayed lower levels with contents of 705.73 μg/kg FW, 697.54 μg/kg FW, 955.13 μg/kg FW, and 854.09 μg/kg FW, respectively. The alcohol contents in both LG01 and LG02 was found to be the highest at 39.89% and 40.30% of the total content, respectively, and they did not contain any sulfur-containing compounds. The aldehyde contents was highest in LG03 to LG09, while the alkane content was highest in LG10. The total sum of the contents was greater than 80%, and the main VOCs in LG01, LG02, LG04, LG05, LG06, LG08, and LG09 were alcohols, alkanes, and aldehydes. The main VOCs of LG03 were aldehydes, alcohols, and furans. Similarly, those found in LG07 were aldehydes, acids alcohols, and alkanes. Finally, the main VOCs in LG10 were alkanes, alcohols, esters, and aldehydes. Most fresh pear varieties had the highest content of esters and alcohols, but the contents and types of VOCs may be altered when processed into pear syrups [9,27]. 

### 3.2. Multivariate Statistical Analysis of VOCs

#### 3.2.1. Common and Characteristic VOCs

**Figure 2:**
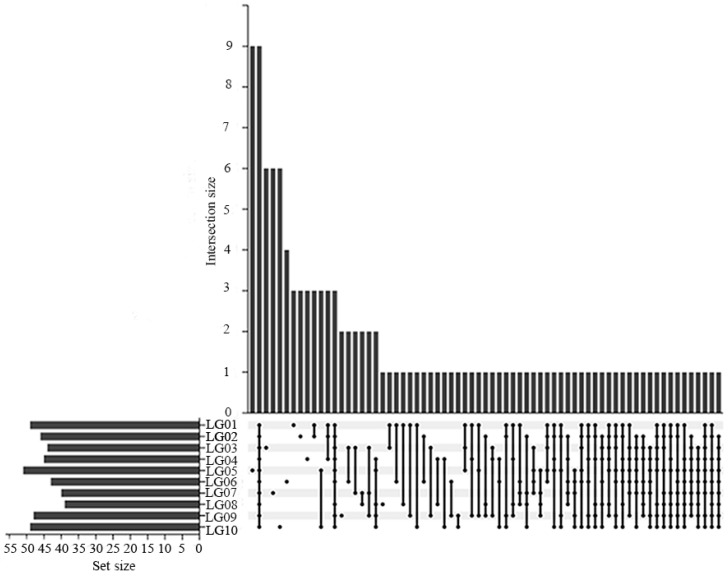
Upset map of VOCs in 10 pear syrups.

Through the upset map analysis (Figure 2), it was determined that ten pear syrups shared a total of nine VOCs, which were 1-Hexanol, 2-ethyl-, Furfural, 3-Pentanone, Butanoic acid, methyl ester, Butanoic acid, 2-methyl-, methyl ester, Hexanoic acid ethyl ester, o-Xylene, and Undecane. The flavor profile is predominantly characterized by sweet, fruity, and flowery [28,29,30,31,32,33]. Additionally, there were a total of 46 characteristic VOCs found in 10 pear syrups (Table 3). They included ten alcohols, four aldehydes, eleven ketones, seven esters, five aromatic hydrocarbons, two alkanes, three sulfur-containing compounds, three terpenes, and one acid. LG01 contained five species, LG02 and LG04 had three species each, LG03, LG07, and LG10 contained six species each, LG05 had the highest with ten species, LG06 had four species, and both LG08 and LG09 had only one species, respectively. The outcomes indicated that the kinds of volatile organic compounds (VOCs) in various pear syrups varied significantly, with each syrup containing a specific proportion of characteristic VOCs. As a result, each syrup had a distinct aroma, making them unique.

#### 3.2.2. Cluster Analysis of VOCs

**Figure 3:**
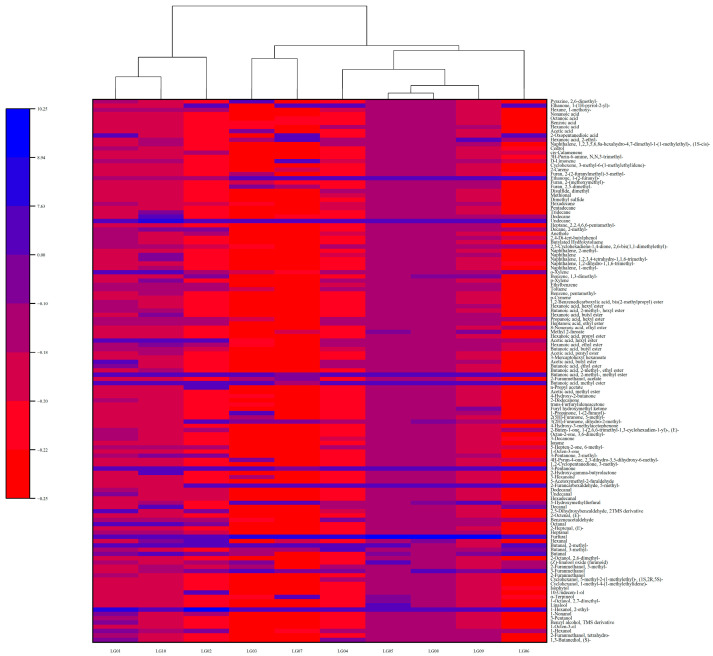
Clustering heat map of VOCs in 10 pear syrups.

The application of cluster analysis is useful for intuitive graphical results representation. Cultivars grouped within the same cluster exhibited comparable characteristics, while those belonging to different clusters showed relatively distinct traits [34]. The clustering heat map of 127 volatile compounds content showed that there were obvious differences between different pear syrups (Figure 3). The outcomes indicated that the aroma profiles of the ten pear syrups were initially categorized into two primary branches, which were further subdivided into three distinct groups. When the samples were divided into three groups, LG01, LG02, and LG10 had similar aromas and were aggregated into one group, and LG04, LG05, LG06, LG08, and LG09 had similar aromas and were aggregated into one group. The heat map clearly distinguishes the VOC concentration of each sample from blue to red.

#### 3.2.3. Difference Analysis of VOCs in 10 Pear Syrups

**Figure 4:**
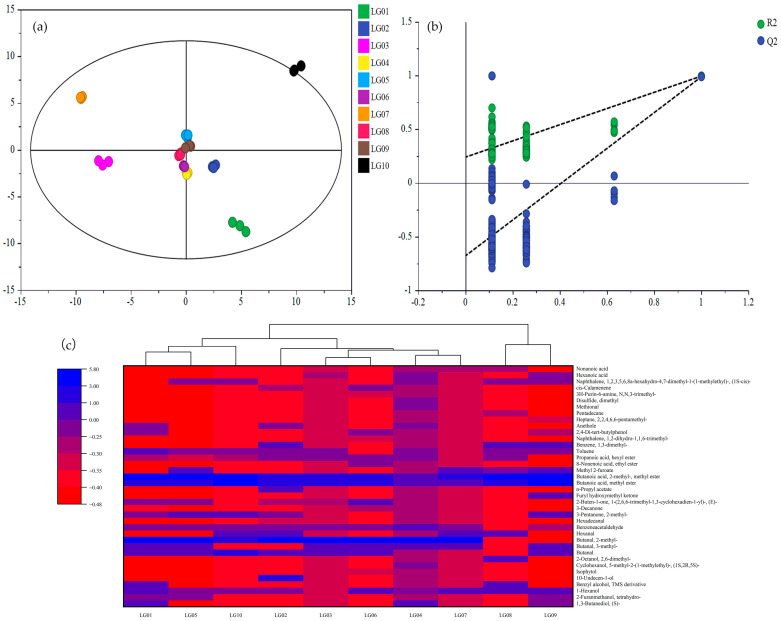
VOCs of 10 pear syrups and an OPLS-DA score chart (**a**), OPLS-DA model validation (**b**), and differential VOC clustering heat map (**c**).

With the VOCs of 10 pear syrups as the dependent variable and different pear syrups samples as the independent variable, the 10 pear syrups were effectively differentiated by OPLS-DA (Figure 4a). LG01, LG03, LG07, and LG10 were obviously distinguished from the other six pear syrups. In the course of this analysis, the independent variable fitting index (Rx2) was 0.982, the dependent variable fitting index (Ry2) was 0.998, and the model prediction index (Q2) was 0.995, with both R2 and Q2 values above 0.5. It was evident that the model’s fitting results were deemed acceptable [35,36]. Following 200 permutation tests, as illustrated in Figure 4b, the intersection of the Q2 regression line with the vertical axis was found to be below 0. Consequently, the results confirm that the model has been successfully validated. It was believed that the results could be used for the differential analysis of pear syrups. To further analyze the VOCs difference of 10 pear syrups, 38 differential VOCs were identified (Figure 4c). Based on the standard of *p* < 0.05 and VIP > 1 (variable importance projection), eight alcohols, six aldehydes, four ketones, six esters, five aromatic hydrocarbons, two alkanes, two sulfur compounds, three terpenoids, and two acids were identified. From the colors, butanoic acid and 2-methyl-, methyl ester were the most prominent aroma components in 10 pear syrups and were also present in strawberry, decoding carob, and *Tetrapleura tetraptera (Thonn.) Taub.* fruit [37,38,39]. Butanal, 2-methyl- was the most prominent VOC in other pear syrups, except LG08 and LG09, and was also present in strawberry and *Tetrapleura tetraptera (Thonn.) Taub.* fruit [37,39]. Butanoic acid and methyl ester were the most prominent VOCs in other pear syrups, except LG01, LG06, and LG07, and were also present in strawberry and decoding carob [37,38]. In addition, LG02 also had a prominent content of 10-Undecen-1-ol with a fresh floral scent [40]. The above results showed that the differential VOC clustering heat map revealed a high abundance of volatile differential VOCs in each pear syrup, indicating that these differential VOCs could be used as a characteristic index to distinguish 10 kinds of pear syrups.

### 3.3. Key VOCs of 10 Pear Syrups

The contribution of aroma components to the food aroma system depended not only on their content but also on their own aroma threshold [41,42,43,44]. OAV could be used to analyze the contribution degree of VOCs of pear syrups. Generally, VOCs with OAV ≥ 1 usually contribute to the overall flavor. OVA values were calculated by each OAV (Table 4). There were 14 compounds with OAV ≥ 1, respectively, for Benzyl alcohol, Butanal, Butanal, 3-methyl-Butanal, 2-methyl-, Hexanal, Octanal, Decanal, 2-Furancarboxaldehyde, 5-methyl-, 1-Octen-3-one, Butanoic acid, methyl ester, Butanoic acid, 2-methyl-, methyl ester, Butanoic acid, ethyl ester, Hexanoic acid, ethyl ester, and Anethole. These VOCs were the key aroma components of 10 pear syrups. Among them, Butanoic acid, 2-methyl-, methyl ester, Butanoic acid, ethyl ester, and Anethole were the common VOCs of 10 pear syrups. LG02 and LG10 accounted for the largest number of key aromas, while LG08 accounted for the least key aromas. LG03, LG04, LG06, LG07, LG08, and LG09 showed the largest OAV with 2-Furancarboxaldehyde and 5-methyl, which contributed the most to the aroma of pear syrups with caramel taste [45]. The key VOCs of LG03 and LG05 were the most similar, but LG05 had an earthy scent. Only LG01 and LG10 were not caramelized, LG01 was mainly sweet and fruity and used for baking, and LG10 was mainly fruity but earthier.

## 4. Discussion

According to the results of the above study, 1-hexanol and 2-ethyl- had the highest VOC content in LG01 and LG02. They have a sweet taste and light floral fragrance, and they are also present in green teas and natto [33,51]. LG01 pear syrup was made of Laiyang pear, which has a light aroma and sweet taste [52]. The key VOC of LG01 was Hexanoic acid and ethyl ester, which is sweet and fruity [31]. LG02 was made of Korla pear, a White Pear variety, which is a specialty of Xinjiang, China. Originating from Xinjiang, China, where it has been cultivated for more than 1300 years, it has garnered the favor of both domestic and international consumers. Its distinct qualities, including a unique fragrance, juiciness, exceptional sweetness, and a particularly special aroma, have contributed to its widespread appeal [53]. The main ingredient of LG02 was a scent of grease, grass, and apple [31], and the key VOC was Hexanal. At the same time, hexanal was identified as a distinctive aroma component of Korla pear, indicating that the choice of raw materials significantly influences the VOCs present in pear syrup [54].

Furfural was the most abundant VOC in pear syrups from LG03 to LG09. It was widely present in tea, virgin olive oil volatilome, and chixiang aroma-type liquor, and it is mainly bready, brown, sweet smell [29,30,31]. The key VOC of LG03, LG04, LG06, LG07, LG08, and LG09 was 1-Octen-3-one, among which LG03 was made of Xuehua pear, which has a single fruit weight of about 400g and rich juice and is often processed into dried fruits and canned products [55]. LG04 was made by Laiyang Cili, which is abundant in Laiyang City, Shandong Province. LG06 was made for Dangshan Suli, which is rich in Dangshan County, Anhui Province, China, and it is a geographical landmark pear product. LG07 was made by Datao Wuming pear and LG08 was made by Yali. Yali is abundant in Hebei Province, China, and is the main pear variety for export. It has a wide cultivated area and an annual output of about 2.6 million tons [56]. LG09 was made by Nanguoli in *Pyrus ussuriensis* Maxim., which is rich in aroma, juicy, and soft after ripening and is a geographical symbol product in Liaoning Province, China. The key VOC of LG05 was Butanoic acid and methyl ester, and it has the odor of apple and cheese aromas [46] and was made from red pear.

Undecane was the highest content VOC in LG10, which mainly existed in the foliar of Hibiscus fragrans Roxburgh [57], but the key VOCs were 2-Furancarboxaldehyde and 5-methyl-, which had a caramel aroma [45]. LG10 was made by Ningling Suli, which is abundant in Ningling County, Henan Province, China. The single fruit weight can reach 800-1000 g or more. When ripe, Ningling Suli is crisp and juicy and sweet and delicious.

It was evident from the above analysis that the VOCs with the highest concentrations in pear syrups were not the key VOCs, which contrasts with Xiao Z.B.‘s findings [58]. Similar to research on soy sauce and strong aroma-type liquors, 2-Pentanol had the highest concentration but an odor activity value (OAV) of less than 1, while Ethyl hexanoate emerged as the most significant aromatic compound. This result aligns with the research carried out by Xiao Z.B. et al. on soy sauce and strong aroma-type liquors, where 2-Pentanol had the highest content but OAV < 1 and Ethyl hexanoate was identified as the key VOC [58].

The raw materials used in pear syrup products on the market mainly consist of White Pear varieties, which are known for their crisp and juicy texture, as well as large single fruit weight. Among these, only LG09 was made by Nanguoli from the *Pyrus ussuriensis* Maxim. due to its strong aroma and softness after ripening. Numerous researchers have discovered that Nanguoli contains nearly 100 VOCs [24], which is a key factor contributing to the abundance of processed products made by Nanguoli.

The VOCs in fruits undergo changes at each stage of processing, and employing low-temperature processing techniques can largely preserve the inherent VOCs of the fruit. However, during hot processing, a substantial alteration occurs in many VOCs due to thermal reactions; specifically, amino acids and reducing sugars participate in Maillard reactions that yield heterocycles or esters among other compounds. This transformation affects both the aromatic substances and their concentrations [59]. Furthermore, certain aldehydes, such as hexanal, exhibit varying odors depending on their concentration levels: at lower concentrations, they impart fruity notes, while at higher concentrations, they produce more pungent flavors [60]. These factors are crucial determinants influencing the aroma profile of processed pear products. 

Pear syrup is a paste product made from the juice of the pear fruit. The color of the pear syrup is yellowish-brown or brown-black, and the taste is sweet. In China, people often drink pear syrup with water, which has the effect of moistening the lungs and relieving cough. Scholars have performed a lot of research on its relieving cough effect [61]. The aroma of processed pear syrup has great changes, and the aroma characteristics of pear syrup are not prominent compared with pear fruit. In the next step, we will study the changes in the aroma of pear fruit after juicing with the extension of heating time and the increase in solid content, find out the key points that cause flavor changes, dig deep into the changes of VOCs, and then change the production process. For example, the low-temperature concentration method retains more pear aroma and lays the foundation for providing better pear syrup products.

## 5. Conclusions

In this study, the author systematically studied the composition and content of VOCs of 10 pear syrups. In total, 127 VOCs were recognized the number of ester and aldehyde compounds was the highest. There were nine common VOCs and forty-six characteristic VOCs of ten pear syrups. By cluster analysis, ten pear syrups were divided into three groups, LG01, LG02 and LG10, LG03 and LG07, LG04, LG05, LG06, and LG08 and LG09, respectively. Using the OPLS-DA method, 38 differentials combined with OAV and 14 key VOCs contributed to the aroma of 10 pear syrups were screened.

Through the above findings, Butanoic acid, methyl ester, Butanoic acid, 2-methyl-, methyl ester, Hexanoic acid, and ethyl ester were found to be the common key VOCs of 10 pear syrups, mainly fruity. 10-Undecen-1-ol, Hexadecanal, n-Propyl acetate, Cyclohexanol, 5-methyl-2-(1-methylethyl)-, (1S,2R,5S)-, Methional, Disulfide, dimethyl, 8-Nonenoic acid, ethyl ester, Naphthalene, 1,2-dihydro-1,1,6-trimethyl-, 3H-Purin-6-amine, N,N,3-trimethyl-, 2-Octanol,2,6-dimethyl-, Furyl hydroxymethyl ketone, Heptane, 2,2,4,6,6-pentamethyl-, Butanoic acid,2-methyl-, and methyl ester were both characteristic VOCs and differential VOCs, with the highest content of 10-Undecen-1-ol, LG02, and LG04 accounting for three kinds, respectively, LG05 and LG09 accounting for two kinds, respectively, and LG06, LG08, and LG10 accounting for one kind. The results showed that the VOCs of 10 pear syrups were rich in diversity, and the aroma of pear syrup made from different varieties of pears was also different. The results of this study could provide a reference for pear processing.

## Figures and Tables

**Table 1:** The 10 pear syrups basic information.

No.	Product Name	Brand	List
LG01	Laiyang pear syrup	Happy domain	Laiyang pear
LG02	Korla pear syrup	Jiangying	Korla pear
LG03	Pear syrup	Huilu food	Xuehua pear
LG04	Laiyang Cili pear syrup	Jian Pu	Laiyang Cili
LG05	Red pear syrup	Wang Jinji	Red pear
LG06	Dangshan pear syrup	Longrui Tangji	Dangshan suli
LG07	Datao Wuming pear syrup	Yancheng Xifeng Datao Wuming pear Co., Ltd. Jiangsu, China	Datao Wuming pear
LG08	Pear syrup	Yafeng	Yali
LG09	Nanguoli pear syrup	Zhenglitou	Nanguoli
LG10	Pear syrup	Xiaolimei	Ningling suli

**Table 2:** Qualitative and quantitative analysis of VOCs in 10 pear syrups.

Compounds Name	Content (µg/kg FW)									
	LG01	LG02	LG03	LG04	LG05	LG06	LG07	LG08	LG09	LG10
Alcohols										
1,3-Butanediol, (S)-	2.66 ± 0.26 b	-	-	6.63 ± 0.12 a	-	-	-	-	1.64 ± 0.18 c	-
2-Furanmethanol, tetrahydro-	0.95 ± 0.20 b	-	-	-	1.51 ± 0.44 b	-	-	-	1.74 ± 0.03 a	-
1-Hexanol	3.42 ± 0.05 bc	4.16 ± 0.16 a	-	4.10 ± 0.32 a	2.30 ± 0.13 d	3.12 ± 0.40 c	3.67 ± 0.06 b	2.99 ± 0.1 c	4.46 ± 0.34 a	2.05 ± 0.26 d
1-Octen-3-ol	-	-	-	-	2.23 ± 0.47	-	-	-	-	-
Benzyl alcohol	3.02 ± 0.21 a	-	-	-	-	-	-	2.85 ± 0.2 a	-	-
3-Pentanol	1.67 ± 0.17	-	-	-	-	-	-	-	-	-
1-Nonanol	3.37 ± 0.76 a	1.73 ± 0.19 b	-	-	-	-	-	-	-	-
1-Hexanol, 2-ethyl-	263.75 ± 2.03 ab	249.66 ± 5.78 abc	230.85 ± 17.22 c	255.67 ± 16.76 abc	237.55 ± 18.70 bc	185.71 ± 17.14 d	272 ± 15.53 a	250.84 ± 19.02 abc	258.20 ± 4.16 ab	269.78 ± 5.23 a
Linalool	-	-	-	-	15.54 ± 0.53	-	-	-	-	-
1-Octanol, 2,7-dimethyl-	-	-	-	-	6.68 ± 0.19	-	-	-	-	-
α-Terpineol	-	-	1.66 ± 0.27 bc	-	5.01 ± 0.24 b	0.23 ± 0.03 c	38.88 ± 3.68 a	-	-	-
10-Undecen-1-ol	-	18.05 ± 0.71	-	-	-	-	-	-	-	-
Isophytol	-	-		-	-	0.22 ± 0.04	-	-	-	-
Cyclohexanol, 1-methyl-4-(1-methylethylidene)-	-	-	-	-	-	-	0.88 ± 0.08	-	-	-
Cyclohexanol, 5-methyl-2-(1-methylethyl)-, (1S,2R,5S)-	-	-	-	1.49 ± 0.04	-	-	-	-	-	-
2-Furanmethanol	0.95 ± 0.20	-	-	-	-	-	-	-	-	-
3-Furanmethanol	-	4.75 ± 0.29 c	119.27 ± 21.63 a	6.80 ± 0.36 c	3.59 ± 0.34 c	46.49 ± 10.75 b	16.98 ± 0.26 c	11.57 ± 0.65 c	4.82 ± 0.57 c	2.89 ± 0.07 c
2-Furanmethanol, 5-methyl-	-	0.75 ± 0.02 d	6.12 ± 0.47 a	2.17 ± 0.31 b	-	1.66 ± 0.03 c	-	0.57 ± 0.05 d	-	0.96 ± 0.13 d
(Z)-linalool oxide (furanoid)	1.71 ± 0.33 c	2.04 ± 0.30 c	11.58 ± 1.49 b	-	18.69 ± 0.36 a	3.05 ± 0.10 c	-	-	-	-
2-Octanol, 2,6-dimethyl-	-	-	-	-	-	-	-	2.37 ± 0.38 a	-	-
Aldehydes										
Butanal	5.09 ± 0.10 c	5.26 ± 0.27 c	7.54 ± 0.61 b	-	5.73 ± 0.21 c	8.37 ± 0.53 a	-	-	7.19 ± 0.17 b	8.82 ± 0.27 a
Butanal, 3-methyl-	2.06 ± 0.11 f	-	5.09 ± 0.09 c	12.81 ± 0.31 a	3.82 ± 0.05 e	5.49 ± 0.34 c	9.84 ± 0.36 b	-	4.49 ± 0.04 d	-
Butanal, 2-methyl-	17.07 ± 0.66 f	62.79 ± 3.61 b	34.83 ± 3.51 d	104.29 ± 2.90 a	22.22 ± 1.33 e	37.83 ± 2.05 d	48.99 ± 0.90 c	-	-	13.68 ± 0.37 f
Hexanal	-	7.11 ± 2.31 a	-	-	-	-	4.53 ± 0.41 a	5.20 ± 0.85 a	-	6.05 ± 0.75 a
Furfural	57.20 ± 4.88 gh	67.52 ± 1.93 g	593.71 ± 44.69 c	400.19 ± 13.82 e	650.18 ± 28.70 b	238.06 ± 18.49 f	704.49 ± 7.60 a	658.83 ± 33.00 b	539.44 ± 23.78 d	19.58 ± 2.03 h
Heptanal	0.36 ± 0.04 bc	0.41 ± 0.05 b	0.17 ± 0.00 e	-	0.28 ± 0.03 cd	-	0.30 ± 0.05 c	0.21 ± 0.02 de	0.31 ± 0.10 c	0.97 ± 0.01 a
2-Heptenal, (E)-	-	-	-	-	1.52 ± 0.30	-	-	-	-	-
Octanal	5.76 ± 0.82 a	3.10 ± 0.71 b	-	-	-	-	-	-	1.28 ± 0.29 c	6.84 ± 0.60 a
Benzeneacetaldehyde	1.57 ± 0.31 b	1.85 ± 0.21 b	0.62 ± 0.04 c	4.91 ± 1.31 a	1.96 ± 0.19 b	1.49 ± 0.23 bc	1.10 ± 0.16 bc	-	1.12 ± 0.15 bc	1.27 ± 0.08 bc
2-Octenal, (E)-	-	-	-	-	2.16 ± 0.19	-	-	-	-	-
2,5-Dihydroxybenzaldehyde, 2TMS derivative	6.78 ± 1.22 a	4.47 ± 1.74 a	-	0.35 ± 0.14 b	-	-	-	-	-	-
Decanal	-	-	-	3.50 ± 0.42 b	-	4.47 ± 0.94 b	-	-	-	15.99 ± 0.67 a
5-Hydroxymethylfurfural	-	-	22.20 ± 3.50 b	3.82 ± 0.30 cd	1.48 ± 0.39 d	3.58 ± 0.35 cd	25.17 ± 2.04 a	4.88 ± 1.04 c	20.23 ± 1.84 b	2.77 ± 0.22 cd
Hexadecanal	-	0.72 ± 0.10	-	-	-	-	-	-	-	-
Undecanal	2.93 ± 0.34 a	0.65 ± 0.17 b	-	-	-	-	-	-	-	-
Dodecanal	1.85 ± 0.63 a	0.49 ± 0.10 b	-	-	-	-	-	-	-	-
2-Furancarboxaldehyde, 5-methyl-	-	23.08 ± 1.18 f	292.07 ± 16.56 b	86.84 ± 7.19 c	53.04 ± 5.38 d	39.55 ± 3.27 e	432.76 ± 8.13 a	58.96 ± 5.20 d	34.72 ± 2.59 ef	-
5-Acetoxymethyl-2-furaldehyde	-	-	-	-	-	-	0.61 ± 0.07	-	-	-
Ketones										
3-Hexanone	-	-	3.88 ± 0.54	-	-	-	-	-	-	-
2-Hydroxy-gamma-butyrolactone	-	-	-	-	-	-	-	-	-	24.92 ± 2.54
3-Pentanone	19.69 ± 0.74 f	22.81 ± 0.59 e	17.31 ± 1.10 g	24.48 ± 0.38 de	26.34 ± 1.00 d	26.62 ± 1.94 d	18.72 ± 0.54 fg	32.84 ± 1.62 c	35.88 ± 0.66 b	40.60 ± 2.64 a
1,2-Cyclopentanedione, 3-methyl-	-	-	0.22 ± 0.05	-	-	-	-	-	-	-
4H-Pyran-4-one, 2,3-dihydro-3,5-dihydroxy-6-methyl-	-	-	12.35 ± 0.07 a	-	-	1.35 ± 0.23 b	-	-	-	-
3-Pentanone, 2-methyl-	1.39 ± 0.14 d	4.05 ± 0.36 b	-	-	3.21 ± 0.22 c	-	-	-	4.34 ± 0.13 ab	4.84 ± 0.47 a
1-Octen-3-one	-	-	-	-	1.36 ± 0.30 a	-	-	-	-	0.15 ± 0.01 b
5-Hepten-2-one, 6-methyl-	2.40 ± 0.37 ab	1.68 ± 0.49 c	-	2.01 ± 0.22 bc	2.87 ± 0.29 a	1.15 ± 0.10 d	-	0.39 ± 0.04 e	-	2.24 ± 0.20 b
Ionone	-	-	-	-	1.63 ± 0.18	-	-	-	-	-
3-Decanone	0.29 ± 0.01 c	-	-	0.36 ± 0.02 b	-	0.53 ± 0.03 a	-	-	-	-
Octan-2-one, 3,6-dimethyl-	0.49 ± 0.19	-	-	-	-	-	-	-	-	-
2-Buten-1-one, 1-(2,6,6-trimethyl-1,3-cyclohexadien-1-yl)-, (E)-	1.42 ± 0.19 c	1.39 ± 0.07 c	0.27 ± 0.04 d	-	2.35 ± 0.10 b	2.87 ± 0.12 a	-	-	-	0.19 ± 0.01 d
4-Hydroxy-3-methylacetophenone	-	-	-	-	-	-	0.87 ± 0.06	-	-	-
3(2H)-Furanone, dihydro-2-methyl-	-	5.60 ± 1.44 c	11.73 ± 1.05 b	1.39 ± 0.17 d	-	1.76 ± 0.25 d	13.81 ± 0.39 a	4.38 ± 0.34 c	1.66 ± 0.09 d	-
2(5H)-Furanone, 5-methyl-	-	-	3.35 ± 0.27	-	-	-	-	-	-	-
1-Propanone, 1-(2-furanyl)-	-	-	32.06 ± 1.64 a	-	-	-	-	-	2.07 ± 0.11 b	-
Furyl hydroxymethyl ketone	-	-	-	-	-	-	-	-	5.19 ± 0.52	-
trans-Furfurylideneacetone	-	-	0.73 ± 0.19	-	-	-	-	-	-	-
2-Dodecanone	0.26 ± 0.11	-	-	-	-	-	-	-	-	-
4-Hydroxy-2-butanone	-	-	0.98 ± 0.09	-	-	-	-	-	-	-
Esters										
Acetic acid, methyl ester	-	-	-	-	-	-	0.75 ± 0.04	-	-	-
n-Propyl acetate	-	7.02 ± 0.37	-	-	-	-	-	-	-	-
Butanoic acid, methyl ester	8.87 ± 0.29 g	18.87 ± 0.06 b	14.98 ± 1.20 d	16.51 ± 0.41 c	19.45 ± 0.61 b	10.19 ± 0.42 f	8.12 ± 0.31 g	13.56 ± 0.48 e	25.32 ± 0.11 a	24.96 ± 0.98 a
2-Furanmethanol, acetate	-	-	7.40 ± 0.51 b	-	-	-	16.04 ± 0.39 a	-	-	-
Butanoic acid, 2-methyl-, methyl ester	16.90 ± 0.69 f	20.80 ± 0.05 cd	17.71 ± 1.20 ef	19.46 ± 0.32 d	23.08 ± 0.55 b	21.97 ± 0.94 bc	19.21 ± 0.48 de	26.47 ± 0.91 a	26.22 ± 2.01 a	27.56 ± 0.41 a
Butanoic acid, 2-methyl-, ethyl ester	-	-	-	-	-	-	-	-	-	0.96 ± 0.14
Butanoic acid, ethyl ester	4.40 ± 0.92 a	0.27 ± 0.04 c	-	2.54 ± 0.06 b	-	-	-	-	-	-
Acetic acid, butyl ester	8.88 ± 0.74 a	-	-	3.39 ± 0.42 b	-	-	-	2.92 ± 0.37 b	3.50 ± 0.18 b	-
3-Mercaptohexyl hexanoate	-	-	-	-	-	-	1.85 ± 0.08	-	-	-
Acetic acid, pentyl ester	-	-	-	-	-	-	-	-	-	1.05 ± 0.12
Butanoic acid, butyl ester	0.56 ± 0.10 c	0.54 ± 0.10 c	-	0.48 ± 0.05 c	-	0.90 ± 0.05 b	-	-	0.66 ± 0.04 bc	2.70 ± 0.38 a
Hexanoic acid, ethyl ester	2.38 ± 0.40 b	2.23 ± 0.25 bc	0.55 ± 0.20 f	1.81 ± 0.11 bcd	0.73 ± 0.10 f	1.09 ± 0.03 ef	0.41 ± 0.10 f	1.50 ± 0.11 de	1.64 ± 0.28 cde	6.35 ± 1.07 a
Acetic acid, hexyl ester	6.53 ± 0.79 b	6.42 ± 0.36 b	1.46 ± 0.25 ef	3.68 ± 0.46 c	0.86 ± 0.10 f	2.74 ± 0.21 d	-	2.70 ± 0.30 d	2.01 ± 0.14 de	8.81 ± 0.59 a
Hexanoic acid, propyl ester	-	-	-	-	-	-	-	-	-	0.25 ± 0.02
Methyl 2-furoate	-	-	-	-	4.45 ± 1.19 ab	-	3.89 ± 0.01 b	0.70 ± 0.07 c	5.15 ± 0.26 a	-
8-Nonenoic acid, ethyl ester	-	-	-	-	-	2.22 ± 0.08	-	-	-	-
Heptanoic acid, ethyl ester	1.74 ± 0.20 a	1.49 ± 0.14 b	-	1.44 ± 0.04 b	-	-	-	1.33 ± 0.14 b	0.72 ± 0.06 c	0.82 ± 0.09 c
Propanoic acid, hexyl ester	0.68 ± 0.04 c	2.28 ± 0.20 a	2.09 ± 0.61 a	1.92 ± 0.48 ab	0.45 ± 0.04 c	1.51 ± 0.20 b	-	1.45 ± 0.45 b	0.26 ± 0.05 c	0.61 ± 0.08 c
Hexanoic acid, butyl ester	-	-	-	-	-	-	-	-	0.91 ± 0.13 b	5.36 ± 0.71 a
Butanoic acid, 2-methyl-, hexyl ester	1.59 ± 0.30 a	-	-	-	-	-	-	-	-	1.54 ± 0.21 a
Hexanoic acid, hexyl ester	0.81 ± 0.17 a	-	-	-	-	-	-	-	0.17 ± 0.01 b	-
1,2-Benzenedicarboxylic acid, bis(2-methylpropyl) ester	0.13 ± 0.02 b	-	0.23 ± 0.05 a	-	0.29 ± 0.06 a	-	-	-	-	-
Aromatic hydrocarbons										
p-Cymene	-	-	-	-	0.66 ± 0.04 a	-	-	-	-	-
Benzene, pentamethyl-	-	-	-	-	-	-	-	-	-	1.38 ± 0.09
Toluene	1.98 ± 0.18 abc	2.30 ± 0.03 a	1.77 ± 0.31 cd	2.32 ± 0.36 a	1.38 ± 0.15 d	-	-	1.66 ± 0.21 cd	2.20 ± 0.27 ab	1.86 ± 0.07 bc
Ethylbenzene	1.26 ± 0.26 b	2.15 ± 0.15 a	-	0.43 ± 0.01 cd	0.48 ± 0.12 c	-	0.22 ± 0.04 d	0.49 ± 0.08 c	0.42 ± 0.11 cd	1.97 ± 0.09 a
p-Xylene	-	-	-	1.90 ± 0.59 b	-	-	-	-	-	3.24 ± 0.06 a
Benzene, 1,3-dimethyl-	-	4.49 ± 0.18 b	-	-	-	-	-	5.58 ± 0.58 a	5.64 ± 0.19 a	-
o-Xylene	5.76 ± 1.34 b	2.69 ± 0.03 c	3.04 ± 0.25 c	3.35 ± 0.03 c	2.99 ± 0.27 c	5.15 ± 0.22 b	3.02 ± 0.17 c	2.91 ± 0.05 c	2.70 ± 0.10 c	7.38 ± 1.29 a
Naphthalene, 1-methyl-	-	-	-	-	0.75 ± 0.09 b	-	-	-	-	2.44 ± 0.41 a
Naphthalene, 1,2-dihydro-1,1,6-trimethyl-	-	-	-	-	-	0.33 ± 0.03	-	-	-	-
Naphthalene, 1,2,3,4-tetrahydro-1,1,6-trimethyl-	-	-	-	-	1.39 ± 0.24 b	-	-	-	-	6.35 ± 0.27 a
Naphthalene	-	-	-	0.56 ± 0.02 d	2.53 ± 0.08 b	-	-	0.59 ± 0.07 d	1.03 ± 0.07 c	4.48 ± 0.27 a
Naphthalene, 2-methyl-	-	-	-	-	0.73 ± 0.02	-	-	-	-	-
2,5-Cyclohexadiene-1,4-dione, 2,6-bis(1,1-dimethylethyl)-	2.23 ± 0.13 a	0.72 ± 0.09 e	1.15 ± 0.40 d	1.25 ± 0.29 cd	0.47 ± 0.06 e	1.62 ± 0.20 b	-	1.48 ± 0.11 bcd	1.57 ± 0.13 bc	0.70 ± 0.01 e
Butylated Hydroxytoluene	0.56 ± 0.01	-	-	-	-	-	-	-	-	-
2,4-Di-tert-butylphenol	1.55 ± 0.23	-	-	-	-	1.04 ± 0.10	-	-	-	1.02 ± 0.07
Anethole	1.84 ± 0.21 b	2.47 ± 0.36 a	-	0.97 ± 0.19 c	-	-	-	-	-	-
Alkanes										
Decane, 2-methyl-	1.70 ± 0.35 bc	4.14 ± 0.03 a	-	-	0.96 ± 0.10 d	1.90 ± 0.49 b	1.51 ± 0.14 bcd	1.12 ± 0.15 cd	1.64 ± 0.54 bcd	4.28 ± 0.67 a
Heptane, 2,2,4,6,6-pentamethyl-	-	-	-	-	-	-	-	-	0.89 ± 0.07	-
Undecane	212.41 ± 27.62 b	111.95 ± 20.30 d	127.75 ± 20.54 d	116.37 ± 15.09 d	141.05 ± 27.57 cd	225.05 ± 15.84 b	183.83 ± 16.44 bc	157.77 ± 9.59 cd	179.56 ± 20.57 bcd	290.22 ± 61.43 a
Dodecane	-	-	-	-	1.41 ± 0.16 b	1.61 ± 0.17 b	2.10 ± 0.18 b	-	1.99 ± 0.50 b	12.76 ± 0.96 a
Tridecane	-	-	-	-	-	-	-	-	-	4.72 ± 0.51
Pentadecane	-	-	-	0.27 ± 0.04 b	-	-	-	0.47 ± 0.02 a	-	-
Hexadecane	0.34 ± 0.06 c	0.45 ± 0.06 bc	1.19 ± 0.17 a	0.55 ± 0.12 b	0.54 ± 0.13 b	-	-	0.47 ± 0.05 bc	0.46 ± 0.02 bc	-
Sulfur-containing compounds										
Dimethyl sulfide	-	-	-	-	3.65 ± 0.29	-	-	-	-	-
Methional	-	-	-	0.84 ± 0.11	-	-	-	-	-	-
Disulfide, dimethyl	-	-	-	4.46 ± 0.25	-	-	-	-	-	-
Furans										
Furan, 2,5-dimethyl-	-	-	8.92 ± 1.10 a	-	-	-	8.33 ± 0.19 a	-	2.86 ± 0.33 b	-
Furan, 2-(methoxymethyl)-	-	-	2.92 ± 0.46 b	-	-	-	3.53 ± 0.08 a	-	2.43 ± 0.18 b	-
Ethanone, 1-(2-furanyl)-	1.71 ± 0.33 e	2.46 ± 0.16 e	214.34 ± 14.24 a	25.12 ± 1.04 d	52.99 ± 2.64 c	27.19 ± 2.89 d	153.88 ± 1.68 b	46.19 ± 5.09 c	54.20 ± 3.48 c	1.26 ± 0.19 e
Furan, 2-(2-furanylmethyl)-5-methyl-	-	-	5.94 ± 0.50 a	-	-	0.50 ± 0.04 b	-	-	-	-
Terpenes										
2-Carene	-	-	-	-	-	-	5.24 ± 0.02	-	-	-
Cyclohexene, 3-methyl-6-(1-methylethylidene)-	-	-	-	-	0.76 ± 0.12	-	-	-	-	-
D-Limonene	0.41 ± 0.02 b	-	-	0.31 ± 0.02 b	2.37 ± 0.23 b	0.91 ± 0.05 b	124.02 ± 5.73 a	0.36 ± 0.03 b	0.43 ± 0.09 b	0.67 ± 0.08 b
3H-Purin-6-amine, N,N,3-trimethyl-	-	-	-	-	-	0.36 ± 0.01	-	-	-	-
cis-Calamenene	-	1.57 ± 0.29 a	-	-	-	1.11 ± 0.23 a	-	-	-	-
Cedrol	0.60 ± 0.14 a	-	-	-	-	0.23 ± 0.01 b	-	-	-	-
Naphthalene, 1,2,3,5,6,8a-hexahydro-4,7-dimethyl-1-(1-methylethyl)-, (1S-cis)-	-	-	-	1.48 ± 0.26 a	1.54 ± 0.08 a	-	-	0.87 ± 0.01 c	1.41 ± 0.04 ab	1.18 ± 0.14 b
Acids										
Hexanoic acid, 2-ethyl-	-	-	7.17 ± 1.29 bc	1.13 ± 0.20 c	1.78 ± 0.52 c	1.63 ± 0.15 c	144.94 ± 13.51 a	-	16.01 ± 0.55 b	0.78 ± 0.11 c
2-Oxopentanedioic acid	9.68 ± 0.52 c					24.44 ± 4.02 b	236.74 ± 4.84 a	3.66 ± 0.38 d		
Acetic acid	-	-	10.34 ± 0.76							
Hexanoic acid	-	-	0.50 ± 0.05 c	1.68 ± 0.06 b	-	-	-	-	2.42 ± 0.45 a	-
Benzoic acid	-	-	0.50 ± 0.04 b	-	-	-	3.18 ± 0.23 a	-	-	-
Octanoic acid	-	-	-	-	-	-	2.22 ± 0.33 a	0.34 ± 0.04 b	-	-
Nonanoic acid							0.47 ± 0.01 a	0.42 ± 0.00 a		
Others										
Hexane, 1-methoxy-	0.84 ± 0.12 b	1.15 ± 0.09 b	-	0.87 ± 0.06 b	0.94 ± 0.05 b	-	-	-	-	2.86 ± 0.57 a
Ethanone, 1-(1H-pyrrol-2-yl)-	-	6.97 ± 1.19 c	-	16.88 ± 0.40 b	-	7.83 ± 0.54 c	58.55 ± 2.28 a	1.67 ± 0.34 d	-	-
Pyrazine, 2,6-dimethyl-	1.29 ± 0.08 b	-	56.05 ± 8.56 a	-	-	-	-	-	-	-

Note: FW means fresh weight; different lowercase letters represent a significant presence at the 5% level; - indicates not detected.

**Table 3:** Differential characteristic VOCs of 10 pear syrups.

No.	Characteristic VOCs
LG01	3-Pentanol, 2-Furanmethanol, Octan-2-one, 3,6-dimethyl-, 2-Dodecanone, Butylated Hydroxytoluene
LG02	10-Undecen-1-ol, Hexadecanal, n-Propyl acetate
LG03	3-Hexanone, 1,2-Cyclopentanedione,3-methyl-, 2(5H)-Furanone,5-methyl-, trans-Furfurylideneacetone, 4-Hydroxy-2-butanone, Acetic acid
LG04	Cyclohexanol,5-methyl-2-(1-methylethyl)-,(1S,2R,5S)-, Methional, Disulfide, dimethyl
LG05	1-Octen-3-ol, Linalool, 1-Octanol,2,7-dimethyl-, 2-Heptenal,(E)-, 2-Octenal,(E)-, Ionone, p-Cymene, Naphthalene,2-methyl-, Dimethyl sulfide, Cyclohexene, 3-methyl-6-(1-methylethylidene)-
LG06	Isophytol, 8-Nonenoic acid, ethyl ester, Naphthalene, 1,2-dihydro-1,1,6-trimethyl-, 3H-Purin-6-amine, N,N,3-trimethyl-
LG07	Cyclohexanol, 1-methyl-4-(1-methylethylidene)-, 5-Acetoxymethyl-2-furaldehyde, 4-Hydroxy-3-methylacetophenone, Acetic acid,methyl ester, 3-Mercaptohexyl hexanoate, 2-Carene
LG08	2-Octanol,2,6-dimethyl-
LG09	Furyl hydroxymethyl ketone, Heptane, 2,2,4,6,6-pentamethyl-
LG10	2-Hydroxy-gamma-butyrolactone, Butanoic acid,2-methyl-,methyl ester, Acetic acid, pentyl ester, Hexanoic acid, propyl ester, Benzene, pentamethyl-, Tridecane

**Table 4:** OVA value and aroma characteristics of the key VOCs in 10 pear syrups.

Compound	Aroma Character	Oder Threshold/(μg/kg) [28,29,31,32,33,43,44,45,46,47,48]	OAV Value									
Benzyl alcohol	Sweet, fruity aromas [31,33,35,46]	3.00	LG01	LG02	LG03	LG04	LG05	LG06	LG07	LG08	LG09	LG10
Butanal	Essence aroma [47]	0.67	1.01	-	-	-	-	-	-	0.95	-	-
Butanal, 3-methyl-	Apple and peach aromas [46,48]	0.10	7.60	7.85	11.25	-	8.55	12.49	-	-	10.74	13.17
Butanal, 2-methyl-	Musty, fermented baking aromas [48]	1.00	20.58	-	50.90	128.12	38.20	54.86	98.39	-	44.87	-
Hexanal	Scent of grease, grass, and apple [30,31,33,35,45,46,47,48]	4.50	17.07	62.79	34.83	104.29	22.22	37.83	48.99	-	-	13.68
Octanal	Fat orange aroma [31,34,35]	2.50	-	1.58	-	-	-	-	1.01	1.16	-	1.34
Decanal	Sweet orange, lemon oil, rose, and wax aromas [31,33,34,35,46]	0.10	2.30	1.24	-	-	-	-	-	-	0.51	2.73
2-Furancarboxaldehyde, 5-methyl-	Caramel aroma [45]	0.50	-	-	-	34.96	-	44.72	-	-	-	159.95
1-Octen-3-one	Earth, mushroom, and metallic aromas [35]	0.01	-	46.16	584.13	173.67	106.09	79.11	865.51	117.93	69.44	-
Butanoic acid, methyl ester	Apple and cheese aromas [46]	7.10	-	-	-	-	272.31	-	-	-	-	29.25
Butanoic acid, 2-methyl-, methyl ester	Fat, apple, and green aromas [49]	5.00	1.25	2.66	2.11	2.33	2.74	1.43	1.14	1.91	3.57	3.52
Butanoic acid, ethyl ester	Apple and pineapple aromas [31]	0.04	3.38	4.16	3.54	3.89	4.62	4.39	3.84	5.29	5.24	5.51
Hexanoic acid, ethyl ester	Sweet and fruity [31]	1.00	109.97	6.70	-	63.41	-	-	-	-	-	-
Anethole	Sweet fennel and licorice [50]	0.50	2.38	2.23	0.55	1.81	0.73	1.09	0.41	1.50	1.64	6.35

Note: - indicates not detected.

## Data Availability

The original contributions presented in the study are included in the article/Supplementary Materials, further inquiries can be directed to the corresponding author.

## Supplementary Materials

The following supporting information can be downloaded at: https://www.mdpi.com/article/10.3390/foods13203223/s1, Figure S1: Photographs of 10 pear syrups; Figure S2: Photographs of 10 pear syrups diluted with water; Figure S3: Total ion chromatograms of GC-MS of 10 pear syrups.

## Abbreviations

VOCs	Volatile organic compounds
HS-SPME	Headspace solid-phase microextraction
GC-MS	Gas chromatography–mass spectrometry
OPLS-DA	Orthogonal partial least squares discriminant analysis
OAV	Odor activity value
DVB/CAR/PDMS	Divinylbenzene, carboxin, and polydimethylsiloxane
EI	Electron ionization
PCA	Principal component analysis
VIP	Variable importance projection
