# Supplementary material for: Identification of Volatile Organic Compounds and Analysis of Aroma Characteristics in Ten Pear Syrups
Source: Foods. 2024 Oct 10;13(20):3223. doi: 10.3390/foods13203223 (PMC11507879; doi:10.3390/foods13203223)
Supplement: Supplementary file 1 [file foods-13-03223-s001.zip › foods-3212131-supplementary.pdf]

Supplementary Material

Identification of Volatile Organic Compounds and Analysis of Aroma Characteristics in 10 Pear Syrups

Yang Wang[1,2,3], Wei Tong[1,2,3]*, Weihui Wang[1,2,3]*, Yanmin Du[1,2,3], Xiaohui Jia[1,2,3], Zhihua Wang[1,2,3], Jianyi Zhang[1,2], Hailong Sun[1]

[1] Institute of Pomology, Chinese Academy of Agricultural Sciences, Xingcheng 125100, China
[2] Key Laboratory of Germplasm Resources Utilization of Horticultural Crops, Ministry of Agriculture and Rural Afffairs, Xingcheng 125100, China
[3] Key Laboratory of Fruits Storage and Processing of Liaoning Province, Xingcheng 125100, China
* Correspondence: tongwei@caas.cn; wangweihui@caas.cn

Table of content

I. Photographs of 10 pear syrups..S3
II. Photographs of 10 pear syrups diluted with water...S4
III. Total ion chromatograms of GC-MS of 10 pear syrups...S5

I. Photographs of 10 pear syrups

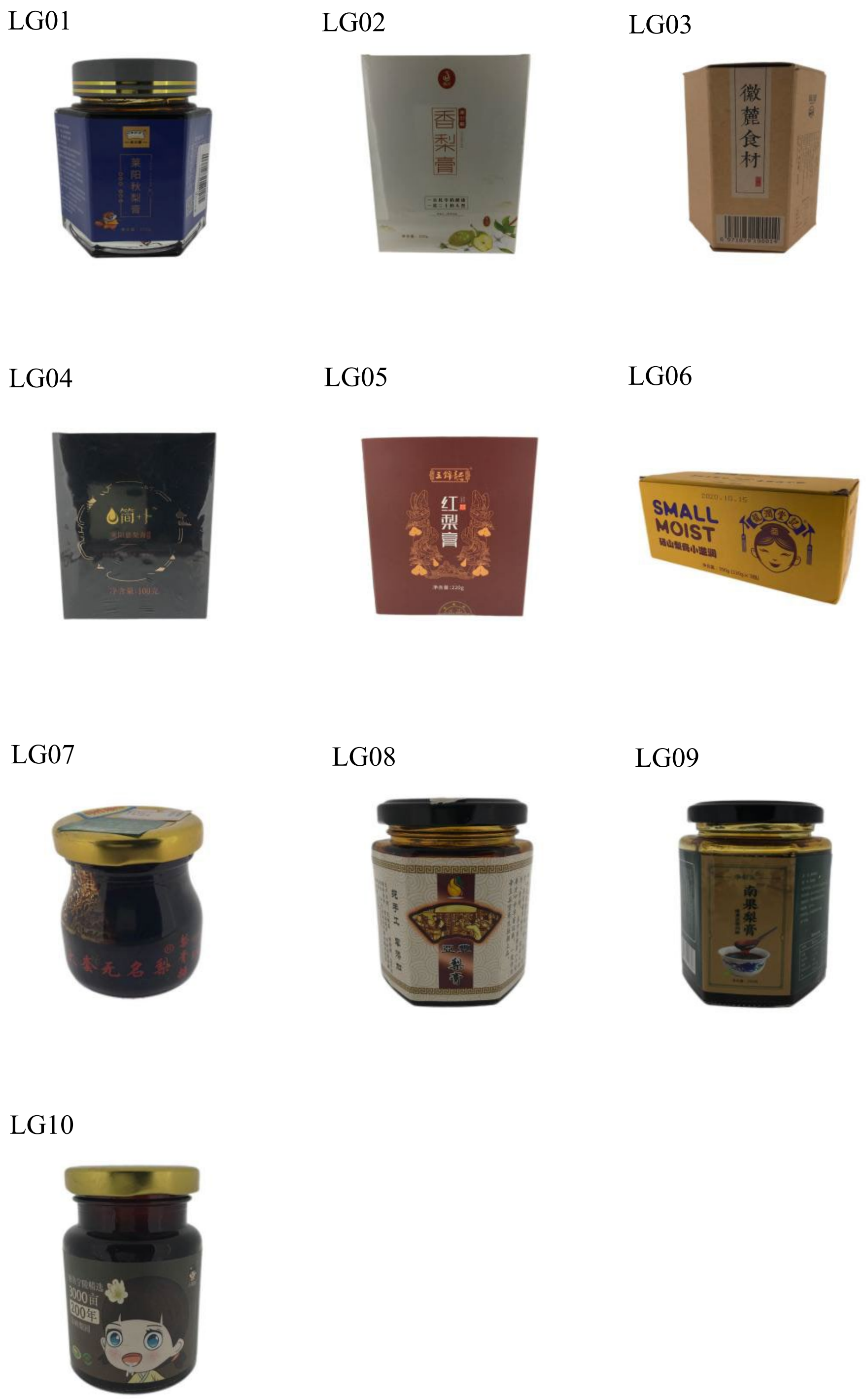

Figure S1. Photographs of 10 pear syrups

II. Photographs of 10 pear syrups diluted with water

The pear syrup itself is very viscous, and the sample needs to be diluted first. Weigh and dilute 4 g pear syrup sample with 20 g purified water. As shown in Figure S2.

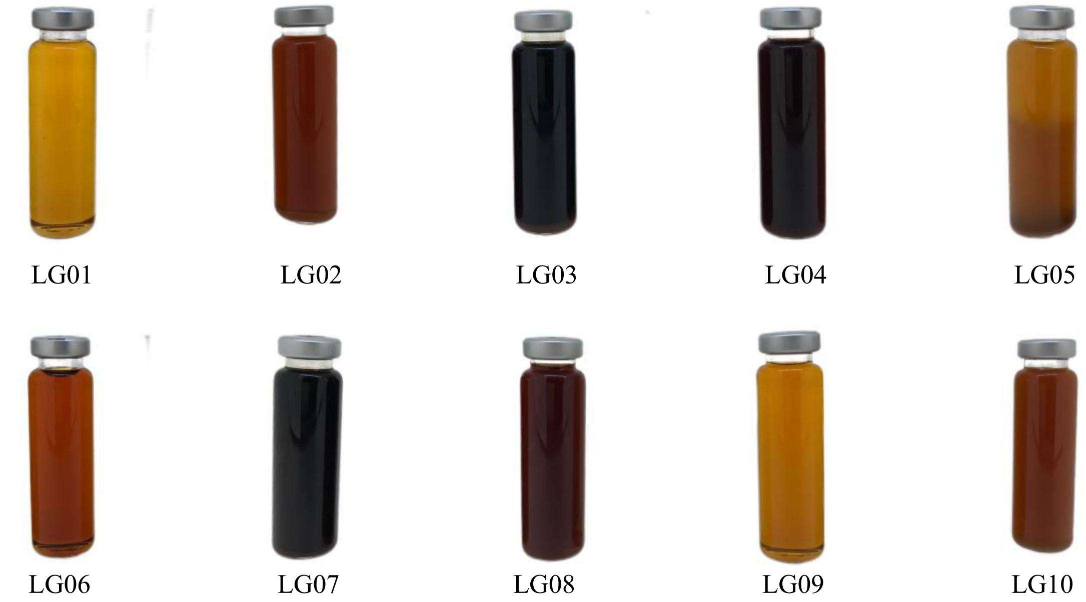

Figure S2. Photographs of 10 pear syrups diluted with water

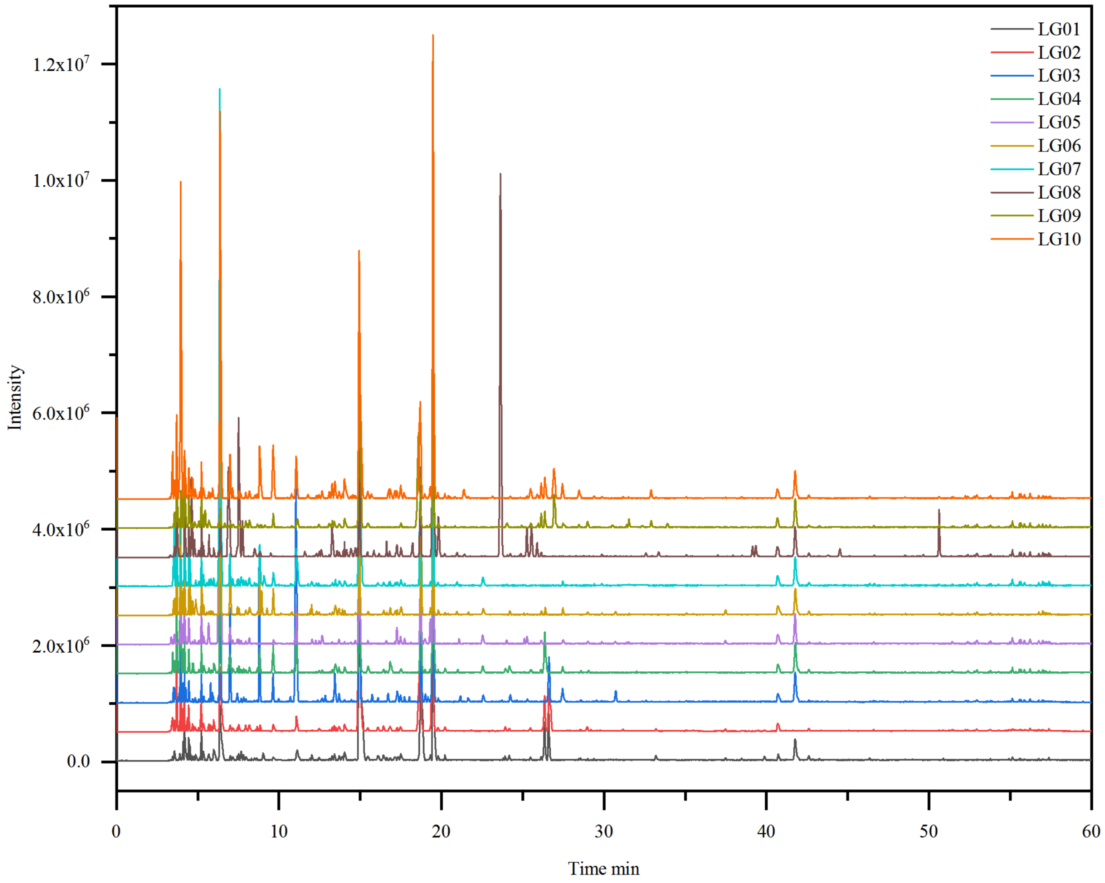

Figure S3. Total ion chromatograms of GC-MS of 10 pear syrups.
